# Cognitive dysfunction in type 2 diabetes patients accompanied with obstructive sleep apnea syndrome

**DOI:** 10.12669/pjms.306.5861

**Published:** 2014

**Authors:** Huanyin Li, Qi Gong, Jinshan Shao, Xueyuan Liu, Yanxin Zhao

**Affiliations:** 1 Huanyin Li, Department of Neurology, Tenth People’s Hospital, Tongji University, 301# Middle Yanchang Road, Shanghai, 200072, China.; 2 Qi Gong, Department of Neurology, Shanghai Minhang Central Hospital, 170# Xinsong Road, Shanghai201199, China.; 3 Jinshan Shao, Department of Neurology, Shanghai Minhang Central Hospital, 170# Xinsong Road, Shanghai201199, China.; 4 Xueyuan Liu, Department of Neurology, Tenth People’s Hospital, Tongji University, 301# Middle Yanchang Road, Shanghai, 200072, China.; 5 Yanxin Zhao, Department of Neurology, Tenth People’s Hospital, Tongji University, 301# Middle Yanchang Road, Shanghai, 200072, China.

**Keywords:** Diabetes, Type 2, Obstructive sleep apnea syndrome, Simple mental state examination scale, Platelet

## Abstract

***Objective: ***To investigate cognitive dysfunction of type 2 diabetes patients accompanied with obstructive sleep apnea syndrome (OSAS), and to analyze its relevant characteristics.

***Methods:*** Total 115 type 2 diabetes patients were divided into OSAS group (O group, n=83) and non-OSAS group (N group, n=32); Physical examination patients (C1 group, n=64) and OSAS patients without diabetes (C2 group, n=47) served as the control group. Apnea-hypopnea index (AHI), nocturnal lowest saturation of pulse oxygen (LSPO2) and simple mental state examination scale (MMSE) were evaluated.

***Results: ***Among diabetes patients, patients with OSAS have lower glycated hemoglobin, platelet count, thrombocytocrit, MMSE score and lowest mean arterial oxygen than non-OSAS patients; cognitive dysfunction state and glycemic control of patients are related to their diabetic duration, and then along with increase of diabetic duration, glycemic control becomes poor, so that cognitive dysfunction becomes more and more obvious.

***Conclusion:*** Along with increased diabetic duration in type 2 diabetes accompanied with OSAS, glycemic control becomes poor, so that cognitive dysfunction more easily occurs. Meanwhile, coagulation function of blood system in OSAS patients with diabetes is impacted to some extent.

## INTRODUCTION

As aging of population becomes more and more obvious, cognitive dysfunction of the elderly population has brought serious spiritual and economic burden to their families and communities. In clinical practice, many factors can cause cognitive dysfunction, including Alzheimer's disease, frontotemporal dementia, corticobasal degeneration and so on; meanwhile because vascular risk factors including hypertension, stroke, diabetes, etc. have its high incidence and universality, the relationship between these factors and cognitive dysfunction has been of great concern recently. Among them, the clinical study that diabetes can cause cognitive dysfunction has been affirmed.^1^^-^^4^

In 2010, a multi-center prospective study (LADIS) in Europe on 639 cases who were 70 years or older had a three-year follow-up and evaluation of cognitive function. After excluding the effect of age, education level, white matter lesions, temporal lobe atrophy and other factors, the researchers have found that diabetes is the only independent risk factor, which can cause cognitive dysfunction, among many vascular risk factors.^1^ Hyperglycemia and hypoxia are two main changes in diabetes frequently associated with several complications, both was also contributing to alter the kinetics, this is a mechanism which might be relevant for diabetes-related complications.^5^ More evidences have demonstrated that among obese patients with type 2 diabetes, more than 75% of patients can have obstructive sleep apnea syndrome (OSAS), and the main pathological change caused by OSAS is chronic tissue hypoxia.^6^

It is thus clear that tissue hypoxia in diabetes patients is inseparable pathological change from hyperglycemia, while hypoxia is an important risk factor of cognitive dysfunction. Therefore, cognitive dysfunction in diabetes patients may be closely associated with hyperglycemia and chronic hypoxia in tissue. This study investigated cognitive dysfunction in type 2 diabetes patients with OSAS through sleep breathing monitoring in type 2 diabetes patients.

## METHODS

***Study subjects: ***The study continuously collected type 2 diabetes patients in the department of internal medicine in Minhang District Central Hospital from October 2012 - July 2013. The disease diagnosis was consistent with World Health Organization diagnostic criteria for diabetes. Polysomnography of 115 cases was completed, including 51 males with mean age (71.60±1.01) and 64 females with mean age (68.75±1.20). This study was approved by the Ethics Committee of our Hospital.

The control group was divided into two groups, of which C1 group came from 64physical examination patients without diabetes in the hospital whose AHI (apnea-hypopnea index) <5 times/hour through monitoring of polysomnography, and C2 group came from 47 patients without diabetes in the department of respiration whose AHI (apnea-hypopnea index) ≥5 times/hour through monitoring of polysomnography.

***General data: ***The recorded data included subjects’ gender, age, fasting blood glucose, glycated hemoglobin, diabetic duration, vascular plaque, platelet count and nocturnal lowest saturation of pulse oxygen (Table-I).

***Sleep breathing monitoring: ***The subjects had begun to be monitored by portable polysomnography from the first night in the hospital. The relevant data were analyzed by machine-specific software. On the first day in the hospital, the sleeps of the subjects were not affected by sedatives, coffee, tea and other factors. The monitoring lasted for more than 6 hours, and the subjects’ AHI, average oxygen concentration and the lowest oxygen concentration were monitored. According to American Sleep Disorders Association (ASDA),^7^ apnea-hypopnea index (AHI) was used as the main observation indicator, specifying that AHI <5 was normal, 5-14 was mild disorder, 15-29 was moderate disorder, ≥ 30 was severe disorder. The sleep breathing monitoring was conducted by specially-assigned persons and the reports were released by qualified doctors in the department of respiration.

***Blood specimen collection: ***On the next morning after the sleep breathing monitoring (overnight fasting for 12h), the subjects’ venous blood was drawn. The automatic hematology analyzer was used to detect blood glucose, glycated hemoglobin, 2-hour postprandial blood glucose, platelet count, platelet distribution width, mean platelet volume, thrombocytocrit, hematocrit and red blood cell distribution width.

***Statistical analysis: ***SPSS 16.0 statistical software was used to treat measurement data. T test was used to compare the measurement data, which was relatively consistent with normal distribution, among the groups; p<0.05 was considered statistically significant. Spearman correlation test and stepwise multiple linear regression analysis were used to analyze the correlation analysis between MMSE and other study indicators; p <0.05 was considered statistically significant.

## RESULTS

There were no differences between the two groups with diabetes of subjects in gender, age, blood glucose, glycated hemoglobin, diabetic duration, carotid artery plaque, sleep-disordered breathing index, platelet count and red blood cell distribution(p>0.05). Among diabetes patients, MMSE scores and glycated hemoglobin in OSAS group were higher than non-OSAS group, but platelet count and thrombocytocritin OSAS group were lower than non-OSAS group. Meanwhile, platelet count and hematocrit in OSAS group were all higher than non-diabetic groups (p<0.05) and MMSE score was highly associated with diabetic duration, glycated hemoglobin, the lowest finger-end oxygen and severity of AHI. It was found that in blood system, patients with obstructive sleep-disordered breathing had decrease of platelet count and could have low thrombocytocrit, and the phenomena were not consistent with relatively high platelet count and elevated thrombocytocrit caused by diabetes itself. Its mechanism may be that hypoxemia induced increase of thrombopoietin, causing drop of platelet count. With MMSE as the dependent variable, multiple linear regression was performed to conclude that MMSE is related to age and lowest finger-end blood oxygen index (Table-II).

**Table-I:** General information.

**Groups**	**Case** **number**	**Age**	**MMSE**	**Blood glucose**	**HBA1C%**	**Duration**	**Plaque**	**LSPO2**	**PLT**	**PLT width**	**PLTvvolume**	**Thrombocytocrit**	**hematocrit**	**Red blood cell width**	**Red blood cell width**
OSAS	83	70.40±10.92	23.39±2.85	6.17±2.41	7.40±1.10	5.04±5.79	8.92±18.71	81.89±4.66	190.47±53.22	13.59±2.46	10.92±1.23	0.20±0.05	37.89±3.48	42.77±2.75	13.20±0.70
nOSAS	32	69.03±12.35	24.90±3.51	5.87±1.54	6.83±0.0.76	3.71±6.71	9.37±17.26	84.44±8.78	212.47±46.95	13.44±2.29	10.79±0.96	0.23±0.04	38.80±2.64	42.56±2.86	13.03±0.65

**Table-II:** Comparison of the indicators between two groups.

	**MMSE**	**HBA1C**	**PLT**	**Thrombocytocrit**	**LSPO2**	**PLT width**	**PLTvolume**	**HCT**	**Erythrocytewidth**	**Erythrocyt** **e** **width**
T value	2.40	3.15	2.05	2.17	2.02	4.39	4.89	5.31	2.09	2.28
P value	0.02	0.02	0.04	0.03	0.04	0.00	0.00	0.00	0.04	0.02

## DISCUSSION

According to the definition of American Sleep Disorders Association, obstructive sleep apnea syndrome (OSAS) is: a patient has repeated episodes of upper airway obstruction during sleep, usually accompanied by reduced blood oxygen saturation; the diagnostic criteria require complaint of daytime sleepiness or excessive sleepiness, and sleep apnea for five times or more per hour is recorded by the polysomnography. Clinically the syndrome is divided into three types: obstructive type, central type and mixed type. In the respiratory laboratory, the obstructive type is the most common type, which is also currently the most intensely studied type. The cognitive impairment of type 2 diabetes patients with OSAS may be associated with the daytime sleepiness, which is due to sleep structural disorder at night, or nocturnal hypoxemia. As degree of sleep-disordered breathing is different, the degree of cognitive impairment is different. The higher the blood glucose level is, the greater the probability of suffering from OSAS. Diabetes can accelerate cognitive dysfunction and is a risk factor of developing senile dementia. Diabetes-related cognitive dysfunction is recognized as another long-term complication of diabetes development.^8^

In addition, OSAS is closely related to neurological diseases. On the one hand, OSAS is one of important reasons causing daytime sleepiness, cognitive dysfunction and certain neurological diseases; OSAS is an important risk factor of cerebrovascular diseases, while it may be closely associated with onset of Alzheimer's disease (AD). On the other hand, neurological diseases are often accompanied with sleep apnea. Clinical studies have found that after acute stroke approximately 32%-71% patients have sleep-disordered breathing, of which 69% to 95% have OSAS. Patients with moderate to severe Parkinson's diseases often have obstructive breathing disorder during the awake and at night. Cognition function damage of OSAS can be impairment of memory, alertness, attention, concentration, judgment, abstraction, reasoning, execution and other functions as well as psychomotor and overall intelligence. Cerebral cortical thinning in cerebral cingulate gyrus, frontal lobe, hippocampus in patients with OSAS^9^ causes study, attention and memory to be worse than the control group. Nocturnal intermittent hypoxia can lead to degenerative changes of the hippocampus in mice, causing spatial learning disorder of the mice, while nocturnal sleep apnea is related to a large number of cognitive and behavioral defects.^10^ Some reports have pointed out that after OSAS patients are given continuous positive airway pressure (CPAP) treatment, attention/vigilance of OSAS patients can be enhanced and executive function and memory function of 50% patients can be improved.^11^ After sleep deprivation ofnormal subjects, attention and memory drop and symptoms of certain psychological changes can be observed. Bedard’s study has found that CPAP treatment can make the night time breathing structure of OSAS patients return to normal and improve daytime alertness; besides two indicators, i.e., planning capabilities and manual flexibility which are indicators significantly associated with nocturnal hypoxemia, all other cognitive functions such as attention, memory and executive ability are improved.^12^ Naegele et al.^13^ have observed a group of 17 patients with OSAS and have found that the change of cognitive function before and after 4 ~ 6 months of CPAP treatment is that except short-term memory span, other cognitive functions related to the frontal lobe such as attention, learning, planning and organizational skills as well as word fluency all recover to normal.

Some studies have reported that more than half of patients with cerebrovascular diseases have OSAS, and OSAS may be an independent risk factor of cerebrovascular diseases because OSAS can cause multi-system damage, for example, OSAS can lead to hypertension, nocturnal hypoxemia and hypercapnia; with extension of the course of the disease, these damages become persistent changes. OSAS may cause increase of renin, angiotensin II and endothelin secretion and increase of sympathetic activity. OSAS allows increase of platelet activity, thus carotid atherosclerotic plaques increase, leading to cerebral blood-supply insufficiency. OSAS can affect inflammation and immune responses by increasing activation of endothelial cells, granulocytes and platelets. These active cells have expressed adhesive molecules and proinflammatory cytokines which may lead to endothelial damage and dysfunction, resulting in the occurrence of cerebrovascular diseases. Moreover, cerebrovascular autoregulation function has changes of structure and function related to age with aging, including β-amyloid (Aβ) accumulation in the matrix of cortical arterioles, neurovascular uncoupling, astrocyte retraction dysfunction, damage to cerebral blood flow, leading to neuronal degeneration, hypoxia and ischemia. At the same time, Aβ deposition in turn exacerbates the decrease of cerebral blood flow, causing brain damage and cognitive dysfunction.^14^ Small vessel lesion caused by OSAS leads to long-term hypo perfusion status of important parts of the cerebral cortex involved in cognitive function and brain tissue which is relatively sensitive to ischemia and hypoxia, especially the hippocampus and limbic temporal lobe. Once necrosis of the hippocampus and limbic temporal lobe occurs, the white matter related to them will have ischemic demyelination and mild cognitive impairment gradually emerges. Next, the annual conversion rate of mild cognitive impairment to dementia is much higher than the age peer group, and actually a relative risk of mild cognitive impairment (MCI) converted to dementia is 9 times higher than normal cognition. Meanwhile, dementia patients have sleep disorders to some extent, and their sleep structural change is just like that of OSAS patients.

This study has found that among hospitalized patients with type 2 diabetes, OSAS incidence rate was 72%, of which frequency of patients with moderate to severe OSAS was 63%. Some studies have found that among patients with type 2 diabetes, the incidence of OSAS is also high. Kosseifiet al.^15^ collected 60 cases of consecutive patients, who were diagnosed of OSAS through polysomnography, and found that 77% of diabetes patients had OSAS. The findings in Japan have also showed that the incidence of OSAS in type 2 diabetes patients reached 77.5%.^16^ All these results have showed that among diabetes patients the incidence of OSAS is relatively high, and meanwhile a variety of related diseases caused by OSAS has been paid more and more attention. Theoretically, type 2 diabetes mellitus is a systemic disease,^17^ thus the respiratory system will be also affected by hyperglycemia and its complications; glucose toxicity and diabetes-related complications can cause respiratory control central or peripheral nervous system lesions, and also affect the muscles related to respiration. Some studies have found that diabetic autonomic neuropathy can lead to autonomic respiratory neuromuscular dysfunction.^18^

Some researchers think that nocturnal hypoxemia is also an important mechanism leading to cognitive impairment of patients with OSAS,^19^ and hypoxemia severity is closely related to degree of cognitive impairment. During sleep of patients with OSAS, many factors can cause narrowing and even obstruction of upper airway, so that hypoventilation, upper airway blockage and apnea occur, resulting in hypoxemia and hypoxia-reperfusion injury.^20^ Hypoxemia can damage endothelial cells and activate blood coagulation system, so that the formed fibrous blood clots are connected to matrix under endothelial cells and the fibrinolytic activity decreases,^21^ causing fibrin deposition and thrombosis to affect cognitive function of patients. Sangal has raised very meaningful questions that OSAS cognitive dysfunction mainly concentrates in the frontal lobe and if untreated, whether patients with abnormal visual P300 will develop into dementia and whether there is correlation between this disease and Alzheimer’s dementia, need to be further explored.

Compared with a set of psychological scales, the MMSE scale saves time and is easily operated, it has become the most commonly used clinical cognitive screening scale. According to the revised domestic MMSE normal standards, illiterate group cutoff value ≤ 19, primary school group cutoff value ≤ 22, junior middle school or above group cutoff value ≤ 26. However, the limitations of MMSE are relatively obvious: it lacks evaluation of executive function and visuo-spatial function, and evaluation of memory is relatively superficial. Therefore, the use of MMSE scale to evaluate cognitive dysfunction in patients with OSAS has some limitations and we hope that evaluation of cognitive function will be further improved.

For type 2 diabetes patients with OSAS, besides blood glucose intervention as soon as possible, CPAP treatment should be given as early as possible if conditions permit,^22^ so as to reduce occurrence of cognitive dysfunction.

## Authors contribution:

**HYL & YXZ** conceived, designed and did statistical analysis & editing of manuscript.

**QG & JSS **did data collection.

**YXZ** did review and final approval of manuscript.

**HYL **takes the responsibility and is accountable for all aspects of the work in ensuring that questions related to the accuracy or integrity of any part of the work are appropriately investigated and resolved.
